# An experimental validation of partial discharge localization using electromagnetic time reversal

**DOI:** 10.1038/s41598-020-80660-z

**Published:** 2021-01-08

**Authors:** Hamidreza Karami, Mohammad Azadifar, Marcos Rubinstein, Farhad Rachidi

**Affiliations:** 1 Electromagnetic Compatibility Laboratory, Swiss Federal Institute of Technology (EPFL), ELL 138, ELL Building, Station 11, Vaud, 1015 Lausanne, Switzerland; 2 Institute for Information and Communication Technologies, University of Applied Sciences of Western Switzerland (HES-SO), Yverdon-les-Bains, Switzerland

**Keywords:** Electrical and electronic engineering, Engineering

## Abstract

The localization of partial discharge (PD) sources is of importance for the monitoring and maintenance of power transformers. Time difference of arrival (TDoA) based methods are widely adopted in the literature for the localization of PDs. Recently, time reversal (TR) was suggested as an efficient means to locate PD sources. As opposed to TDoA, which needs at least 4 sensors, TR is able to locate PD sources in power transformers with only one sensor. Moreover, it needs neither line-of-sight wave propagation from the PD sources to the sensor nor time synchronization. In this study, we present for the first time an experimental demonstration of the ability of the TR process to locate PD sources. A typical TR process includes three steps: (1) recording the PD-emitted field by a sensor, (2) time reversing and back injecting the signal into the medium, (3) using a proper criterion to obtain the focusing point which corresponds to the location of the PD source. In this work, we present a laboratory setup in which steps one and two are performed experimentally, both in the frequency and in the time domain. The obtained peak electric field value is used as a criterion in the third step. It is found that the accuracy of the proposed method is better than 2.5 cm in a transformer tank model with dimensions 73 × 73 × 103 cm^3^. The effects of the presence of scatterers such as transformer windings are also investigated experimentally and found not to affect the location accuracy of the method.

## Introduction

Source localization has many applications in medicine^1,2^, acoustics^3^, electromagnetics^4^, complex networks^5^, etc. In electrical engineering, partial discharges (PDs) are one of the important sources of damage and degradation of the dielectric materials in transformers^6^. The failure of power transformers may cause catastrophic effects in power networks. Early detection and localization of the PDs will lead to much lower power transformers maintenance costs and to an increase in the reliability of power networks.

PDs emit both pressure (30–300 kHz) and electromagnetic (300 MHz–3 GHz) waves in the surrounding medium^6^. Time difference of arrival (TDoA) based methods are commonly used to provide 3D localization of the PD in power transformers^7^. However, a precise determination of the onset time of the arriving signals, which is required in TDoA methods, is problematic due to noise. In addition, the performance of TDoA deteriorates when the transformer winding is located between the sensor and the PD source. Moreover, TDoA-based techniques need at least 4 time-synchronized sensors to operate^3,8–10^.

Recently, the concept of time reversal (TR) cavity presented by Fink and coworkers in^11,12^ was used to localize an electromagnetic interference (EMI) source using a single sensor^13^. The main difference between TDoA-based approaches and TR based methods is the fact that TDoA methods use only the information on the arrival time of the wave, while TR based techniques use the overall waveform features. More recently, a time-reversal based method to localize PD sources was proposed^14^ and its efficiency was demonstrated through numerical simulations applied to several test cases. The proposed method was found to be able to perform 3D localization of PD sources using only one sensor even in the presence of transformer windings, a practical scenario in which the application of TDoA-based methods is problematic.

In this paper, we present for the first time an experimental test aiming at assessing the ability of the electromagnetic time reversal (EMTR) technique to localize PD sources in the presence of the transformer windings. To do this, the PD source is emulated by a single monopole antenna inside a metallic cavity. To record the PD signal, another monopole antenna is used. The general procedure described in^14^ to locate PD sources will be explained later.

It should be noted that to model the propagation in the forward and backward steps, the response of the system (scattering parameter between the antenna and the sensor) is measured in the frequency domain using a vector network analyzer (VNA). We also considered the presence of a metallic object which can be representative of the transformer winding. In order to validate our frequency domain procedure using the VNA, another measurement was performed in the time domain using a wideband oscilloscope and a comparison between the frequency and the time domain results is presented.

To the best of the authors’ knowledge, the novelties of the paper can be considered as follows:

1. The experimental validation of the TR technique inside the 3D cavity (7λ_min_ × 7λ_min_ × 10λ_min_) in the frequency range of 30 kHz-3 GHz in the frequency domain and without any modulation technique.
2. The 2D and 3D numerical validation of the TR technique by considering a 2D and a 3D rectangular cavity structure including multiple objects. The effects of the frequency range on the efficiency of the proposed method is investigated.
3. Single sensor localization of a PD source inside the 3D-cavity (7λ_min_ × 7λ_min_ × 10λ_min_) and experimental investigation of the effects of the presence of an object on the localization accuracy.

## Methods and results

### PD source localization using EMTR: numerical validation

To illustrate the application of the TR technique to locate PD sources in power transformers, we will consider two case studies. In the first case study, a power transformer is modelled using a 2D Finite-Difference Time-Domain (FDTD) technique. The FDTD method solves Maxwell’s equations in transverse magnetic modes. In the second case study, the commercial CST-MWS software is used as a transient solver to simulate the propagation of the electromagnetic wave inside a 3D model of a power transformer tank. The CST-MWS solver is based on the Finite Integration Technique (FIT) and it solves the Maxwell’s equations in their integral form.

The TR algorithm used to validate the PD source localization in 2D and 3D cases can be summarized as follows: In the first step, the electric fields due to the PD source are recorded using the sensor. This step is referred to as the forward propagation step. Then, the recorded signal is time reversed and back injected from the location of the sensor into the medium. This step is referred to as the backward propagation step. Finally, a suitable criterion such as the minimum entropy^15^ or the maximum electric field^16^ is used to locate the PD source.

Some or all the steps in the TR algorithm can be implemented equivalently either in the frequency domain or in the time domain. In this paper, both implementations are considered.

### 2D power transformer model

**Figure 1:**
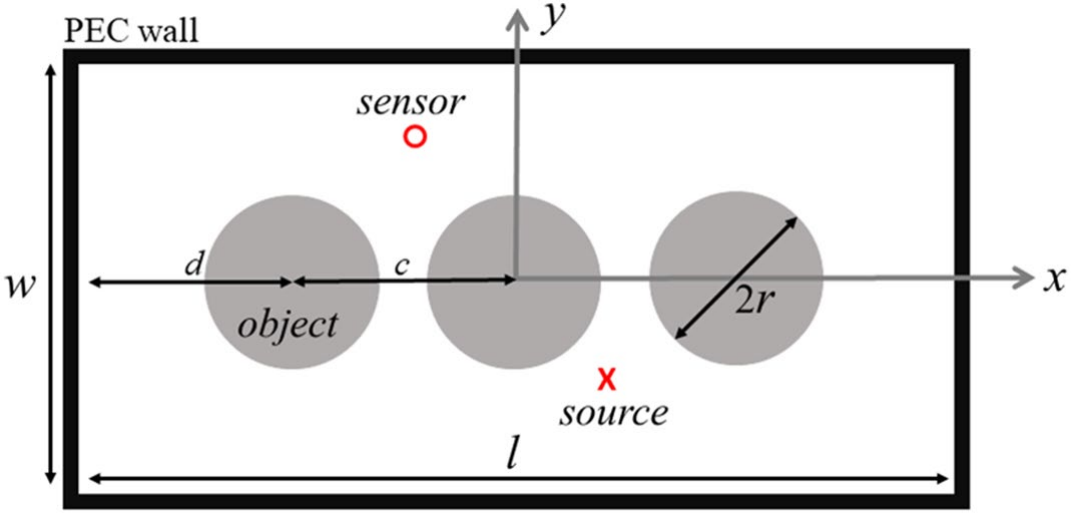
Geometry of the 2D power transformer model, including a metallic tank (thick black PEC frame), source, sensor, and three windings. The tank is symmetric along the x and y axes. *d* = 250 mm, *c* = 250 mm, and *r* = 100 mm. *w* = 0.5 m and *l* = 1 m.

A 2D model of a power transformer including windings^14^ is shown in Fig. 1, which includes one PD source and one sensor. The tank of the transformer is assumed to be a perfect electric conductor (PEC) with dimensions *w* × *l* (0.5 × 1.0 m^2^), which acts as a 2D cavity. The horizontal distance between the center of each one of the outer cylinders and the walls of the tank is 250 mm (*d* in the figure) and the distance between the centers of the contiguous cylinders is 250 mm (*c* in the figure). The radius of each cylinder is 100 mm. The presence of the transformer tank walls leads to an infinite number of paths from the PD source to the sensor, as demonstrated in^13^. In the backward propagation step, the wave converges back to the source location through each of the infinite paths. In other words, a TR cavity can provide us a TR mirror^13,17^. Because of the presence of a surrounding cavity, we chose the maximum field amplitude as the criterion to identify the focal spot. Compared to the minimum entropy criterion, the maximum field criterion leads to a reduction in the computational burden since it does not require the evaluation of the entropy at each time step in the back-propagation step^15,18^.

In the forward-time step, a Gaussian pulse with a bandwidth of 3 GHz is used as the PD source as shown in Fig. S1 (in the Supplementary Information) and the field at the location of the sensor is recorded. The coordinates of the sensor and the source are, respectively, (−0.1 m, 0.15 m) and (0.1 m, −0.15 m), with the middle winding in between and, therefore, with no direct path between them. This situation is very challenging for the other localization methods, like TDoA which requires line of sight between the source and the sensor. The origin and the axes of the selected coordinate system can be seen in Fig. 1. The grey circles show the windings of the power transformer inside the tank, which are assumed to be PEC. In accordance with the procedure described earlier, the signal recorded by the sensor in the forward step is time reversed and back injected into the same medium.

**Figure 2:**
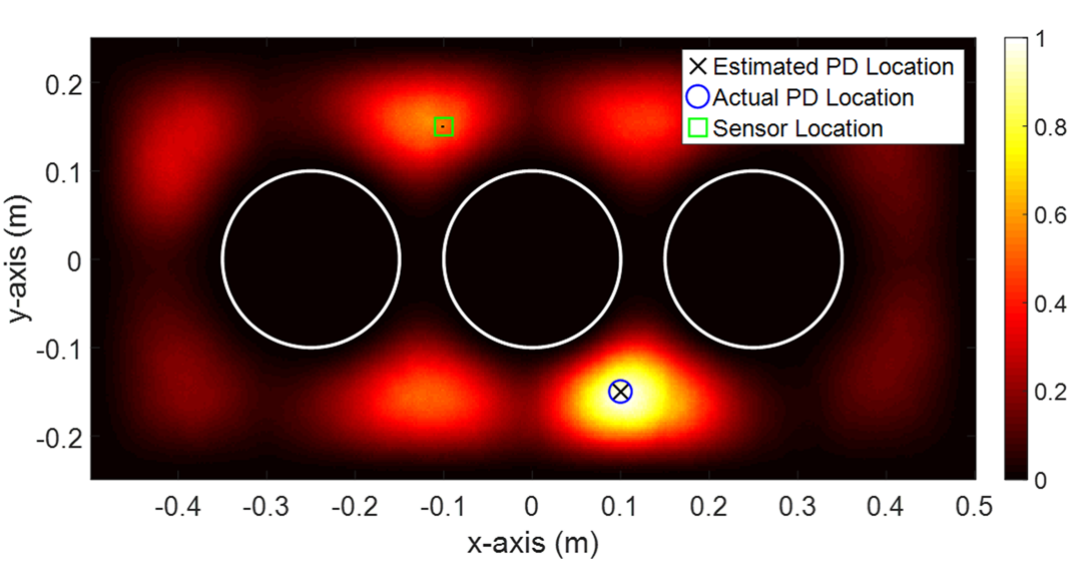
The Distribution of normalized maximum z-component of the electric field intensity over the computational domain. The blue circle and the black cross represent the actual and the estimated locations of the source, respectively. The black point with a length of 1 cell at the location of the sensor (inside the green square) shows the mask filter that is used in this simulation.

The time step of the simulations is 5.3 ps. The number of time steps in the simulations is 6000. The computational domain is meshed using equally spaced square mesh cells with a length of 2.5 mm. The normalized z-component of the maximum electric field intensity over the computational domain for the 3 GHz excitation is shown in Fig. 2. As can be seen, the maximum electric field intensity criterion allows the accurate estimation of the location of the PD source. It should be noted that a square mask was used to ignore the values of the electric field around the sensor. In the considered simulations, the size of the mask was 2.5 × 2.5 mm^2^. A blue circle, a black cross, and a green square show the location of the actual PD source, the estimated PD source, and the sensor, respectively. The error between the estimated and the actual PD source locations is zero for the 3 GHz bandwidth PD source.

**Figure 3:**
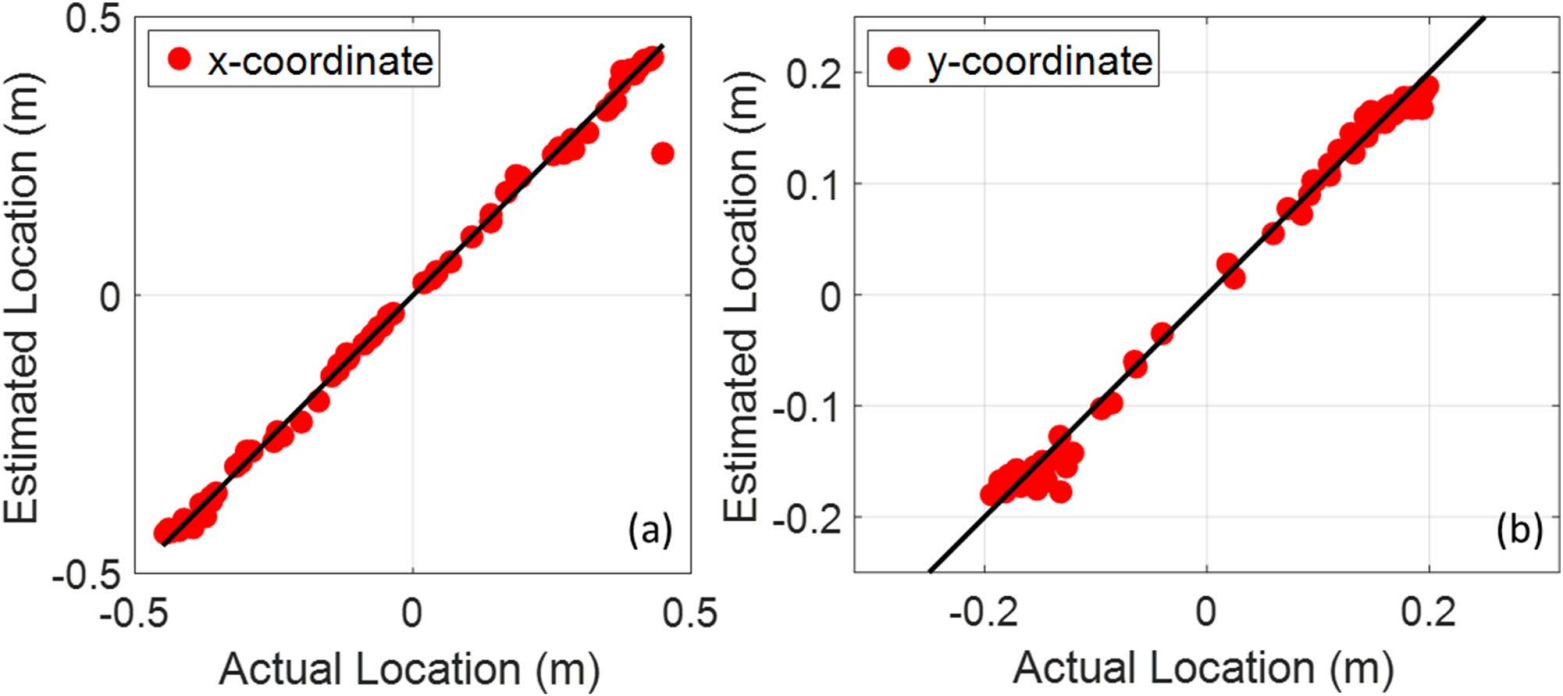
The estimated location versus the actual location of PD sources for the 2D transformer model shown in Fig. 1. The number of Monte-Carlo simulations is 100, (**a**) x-coordinate, (**b**) y-coordinate.

In order to evaluate the performance of the TR algorithm to locate the PD sources, a Monte-Carlo simulation was performed. Monte Carlo simulations were carried out in the following way. First, one hundred locations for the PD sources were generated using a random function with a uniform probability distribution over the computational domain shown in Fig. 1. It should be noted that the locations closer than λ_min_/2 to the transformer winding and tank were removed from the set. As discussed in^19^, PDs closer than about λ_min_/2 to the transformer tank or windings are not accurately localized using the proposed method. Second, the proposed method is applied to localize each PD source. The estimated locations using the proposed algorithm versus the actual locations of the PD sources for the x- and the y- coordinates are shown in Fig. 3. The sparsity of the y-coordinates between −0.15 and 0.15 m is due to the presence of the winding in this range. As we can see in Fig. 3, the accuracy of the proposed method is quite remarkable. The mean, median, and standard deviation of the distance between the real and estimated values are 17.5 mm, 15.1 mm, and 22.7 mm, respectively. Fig. S2 shows the real locations of the PD sources (black filled circles) and the ones estimated by the proposed method (red crosses). The blue-solid lines show the difference between the estimated and the actual locations of the PD sources. As we can see in Fig. S2, one of the PD locations (in the bottom right corner) has not been accurately located. The reason behind this large error is its vicinity to the tank wall (exactly λ_min_/2) (see^19^ for more discussion).

### 3D power transformer model

**Figure 4:**
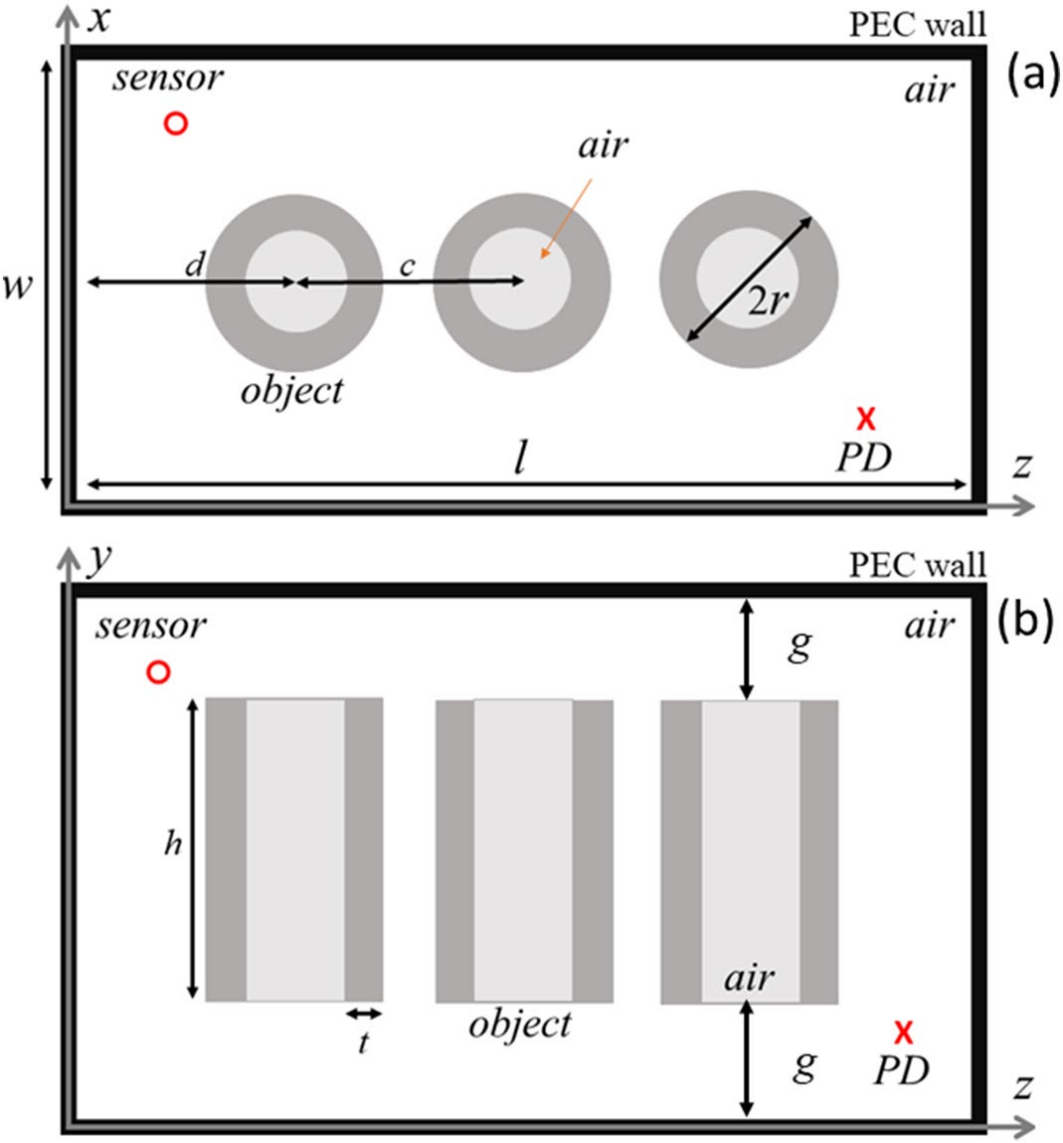
Geometry of the 3D power transformer model, including a metallic tank (thick black PEC frame), a PD source (red cross), a sensor (red circle), and three windings. The tank is symmetric along *xoy* and *xoz* planes. *d* = 250 mm, *c* = 250 mm, *r* = 100 mm, *h* = 300 mm, and *t* = 20 mm.

Figure 4 shows the top and side views of the considered 3D model of a power transformer tank with dimensions 0.5 × 0.5 × 1.0 m^3^. The three unfilled metallic cylinders are used to model the windings. The materials considered for the tank and the winding blocks are steel (σ = 7.69e6 S/m) and copper (σ = 5.8e7 S/m), respectively. The transformer tank walls have a 10 mm thickness. The horizontal distance between the center of each one of the outer cylinders and the walls of the tank is 250 mm (*d* in the figure) and the distance between the centers of the adjacent cylinders is 250 mm (*c* in the figure). The vertical distance between the windings and the upper and lower tank walls is 100 mm (*g* in the Fig. 4b). The outer and inner radii of each object are 100 mm and 80 mm, respectively. The wireframe and solid view of the 3D model of the transformer designed in the CST-MWS software are shown in Figs. S3 and S4. Figure S3 shows the locations of the ports used to sense and excite the 3D transformer model.

We have considered three case studies, summarized in Table 1. A single PD source is considered in Case 1 and two PD sources are considered in the remaining two. The two PD sources in Case 2 are simultaneous and, in Case 3, two non-simultaneous PD sources are considered. The effects of different amplitudes are also investigated in Case 3. For all cases, we used one sensor 50 mm above the tank floor to locate the PDs. The coordinates of the sensor and the PD sources are given in Table 2.

**Table 1:** List of the case studies considered in the paper and the resulting location error.

Case studies	PD source(s)	Location error (mm)
CS #1	PD2	4
CS #2	PD1 & PD2	6 (for PD1) & 5 (for PD2)
CS #3	PD2 & PD3	3 (for PD2) & 4 (for PD3)

**Table 2:** Location of the considered PD sources and single UHF sensor inside the power transformer tank.

PD or S	Location (x, y, z) mm
PD1	(250, 250, 100)
PD2	(150, 250, 100)
PD3	(250, 250, 250)
S	(100, 50, 900)

Dipole antennas were used to model the PD sources inside the transformer tank. The waveform used to excite the dipole antennas was a Gaussian pulse with 1, 2, or 3 GHz frequency bandwidth as shown in Fig. S1. With reference to Table 2, PD1 and PD2 are located outside the windings while PD3 is inside the left winding. Only one UHF sensor is placed inside the tank (S in Table 2) at a location recommended by standards, namely at the bottom as they are assumed to be inserted through the standard drain valve. In all the simulations, the length of the dipole antenna representing the UHF sensor is considered to be 20 mm.

Three copper cylinders, as depicted in Fig. 4, are used to model the windings of the transformer. Note that, as mentioned earlier, the location of PD3 is within the winding and there is no line-of-sight path to the sensor, a scenario which is quite challenging for classical PD localization approaches such as TDoA.

In accordance with the procedure described in Section I, the signal recorded by the sensor in the forward step is time reversed and back injected into the same medium. All the steps are numerically simulated using the CST-MWS software. The focal spots were determined using the maximum electric field power at all the time steps. It should be noted that the sensor location is not considered in the maximum electric field power calculations^16^.

**Figure 5:**
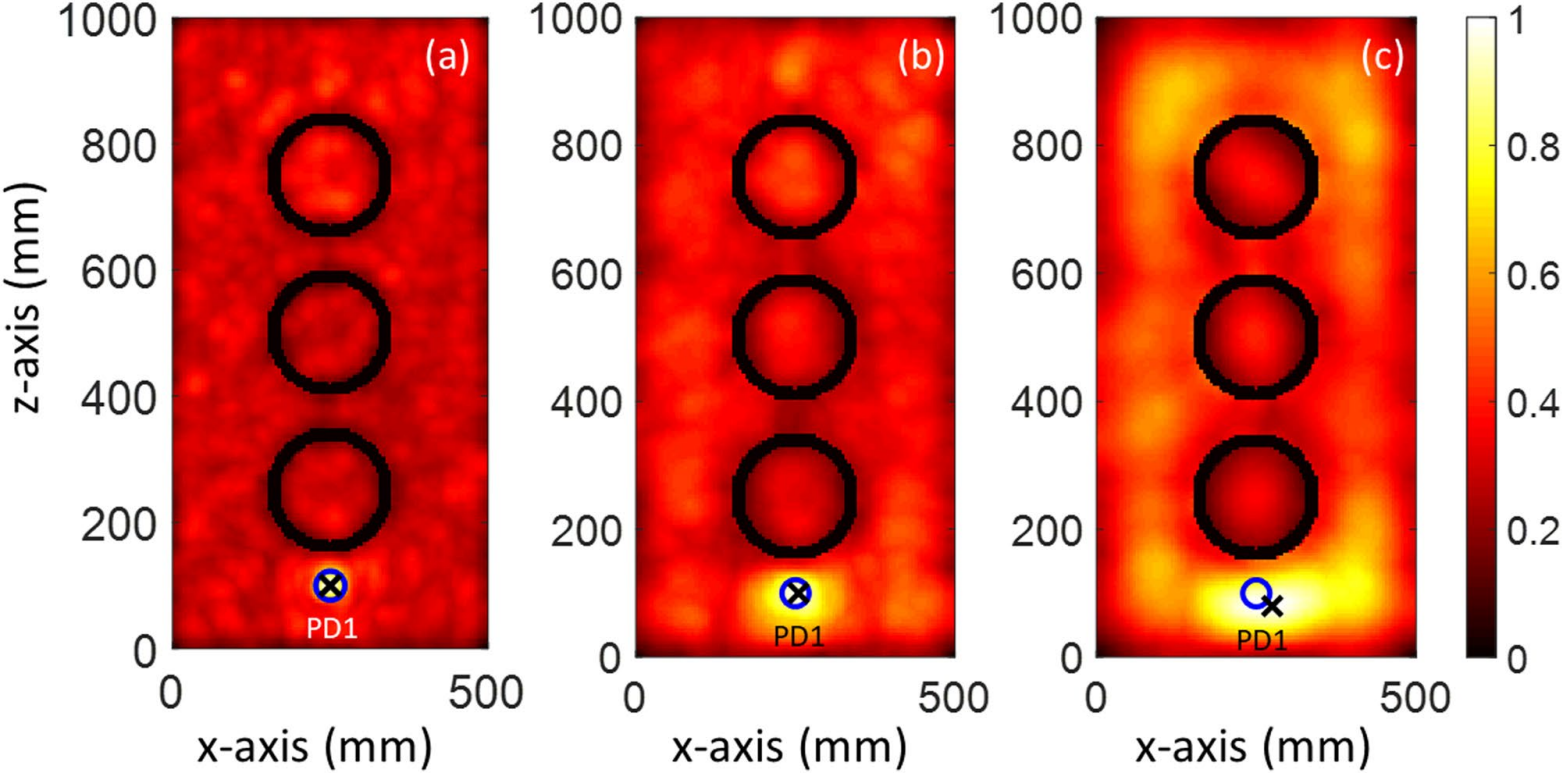
The Distribution of the normalized maximum y-component of the electric field intensity in the *x-z* cut plane (CS #1). The blue circle and black cross show the actual and estimated location of the PD1 source, respectively. The bandwidth of the PD1 source is (**a**) 3 GHz, (**b**) 2 GHz, and (**c**) 1 GHz.

In the first case study (CS#1), three Gaussian pulses with 1 GHz, 2 GHz, and 3 GHz bandwidth, shown in Fig. S1, were considered to excite the PD1 source. Figure 5 displays the normalized distribution of the maximum y-component of the electric field in the *x*–*z* cut plane at *y* = *y*_max_. As can be seen in Fig. 5, increasing the considered bandwidth for the PD1 source leads to a smaller focal spot for the proposed method. As a result, the location error will decrease with increasing bandwidth. The location errors for 1, 2, and 3 GHz bandwidth of the PD1 source are 32 mm, 5 mm, and zero, respectively. All the location errors are 3D distances and are smaller than λ/10, λ being equal to 100 mm (corresponding to the maximum operational frequency in 3 GHz). The EMTR method is able to focus on the source, with a focal spot of λ/2 for the conventional EMTR and smaller (e.g., λ /30) in a multipath environment or applying multiresolution techniques^20–22^. Therefore, by increasing the bandwidth, the minimum wavelength is decreased, resulting in a smaller focal spot (for more details, see references^20–22^).

Furthermore, the number of excited modes increases with the bandwidth. These modes are uncorrelated and contribute incoherently except at the focal point^23^. Thus, an increase in the number of modes resulting from a wider bandwidth leads to a smaller focal spot.

It should be noted that the overall maximum over the whole time and space coincides with the location of the sensor and it should therefore be ignored. Since the primary knowledge of the location of the sensor is available, its location can therefore be disregarded in the maximum electric field evaluation. The second overall maximum corresponds to the estimated location of the PD source (PD1). As shown in Fig. 5, the locations of the windings can be identified as the zones with close-to-zero fields (black circles). The presented results confirm that the proposed TR method can successfully locate a PD source even considering the transformer windings. In other words, the presence of large scatterers such as windings does not degrade the performance of the proposed method. In addition, the results presented in Fig. 5 show that there is a trade-off between the measurement frequency bandwidth and the location accuracy of the proposed method. By increasing the frequency bandwidth of the system recording the signal in the forward step (measurement phase), the location accuracy of the method will be improved. However, the cost of the measurement system will increase because of the required higher sampling frequency rate. Note that the location accuracy of the proposed method is still acceptable for a frequency bandwidth limited to 1 GHz.

**Figure 6:**
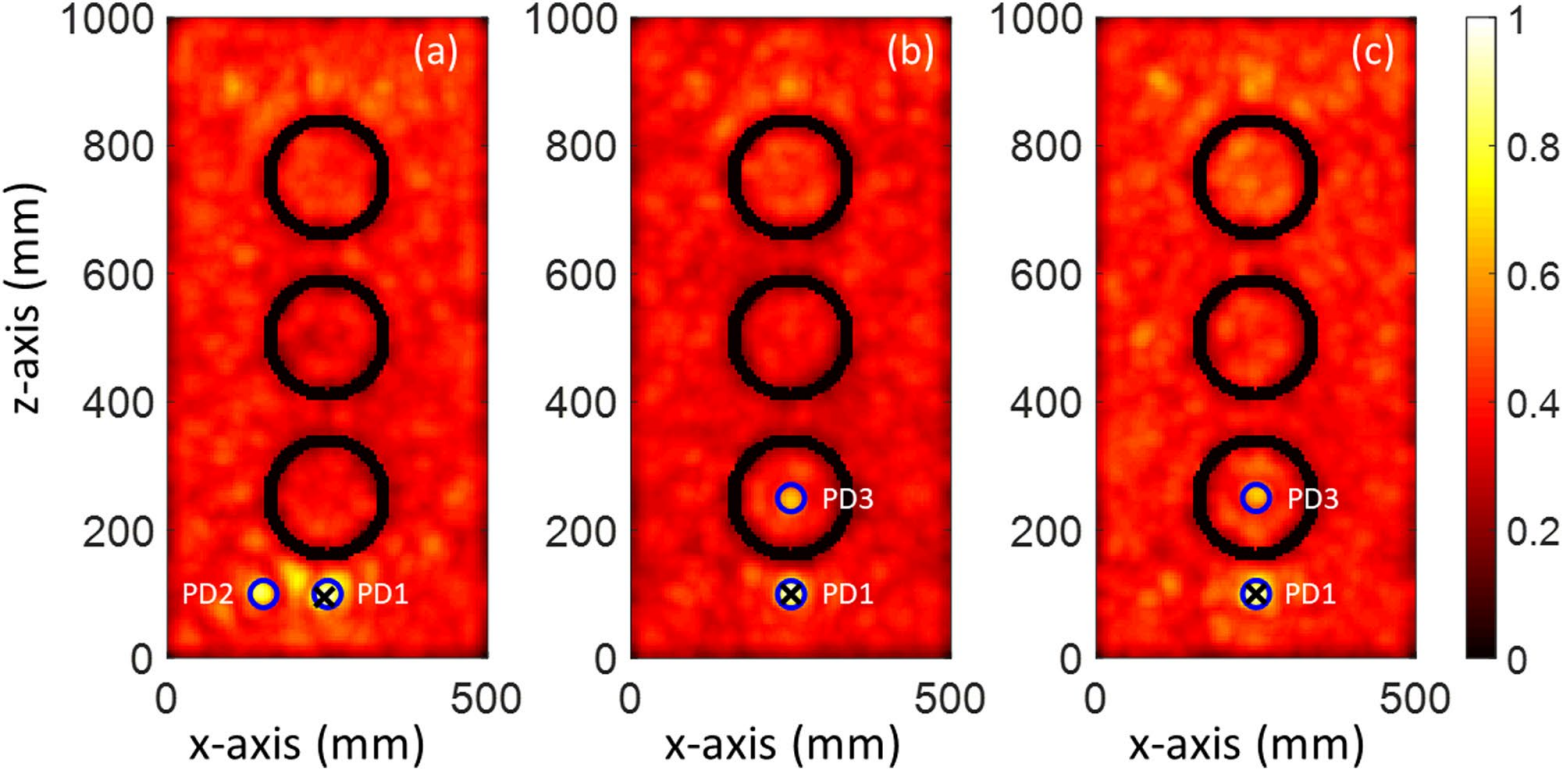
The Distribution of the normalized maximum y-component of the electric field intensity in the *x–z* cut plane (CS #1). The blue circles and black cross show, respectively, the actual locations of the PD sources and of the maxima of the electric field intensity. (**a**) Scenario based on CS#2, (**b**) scenario based on CS#3 with simultaneous PD sources, and (**c**) scenario based on CS#3 with non-simultaneous PD sources.

In a real power transformer, multiple PD sources might occur inside the tank. The second case study shows the ability of the proposed method to locate multiple simultaneous sources using only one sensor. In the second case study, the location of the sensor (S) is kept as that of CS#1. The locations of the PD sources are given in Table 2. Figure 6a shows the distribution of the normalized maximum of the y-component of the electric field intensity over the whole simulation time inside the transformer tank in the *x–z* cut plane (*y* = 250 mm) for CS#2. The blue circles and the black cross show, respectively, the actual locations of the PD sources and the maximum field intensity of the plane. In the figure, the locations of the metallic cylindrical windings can be clearly identified as they correspond to zones of minimum field. The 3D location errors for CS#2 are estimated to be 6 mm and 5 mm for PD1 and PD2, respectively. It can be seen that the proposed method is capable of locating multiple simultaneous sources with high accuracy, considering the presence of the windings. For better illustration, the distribution of the y-component of the maximum electric field intensity in the *x–y* (*z* = 100 mm) and *y–z* (*x* = 250 mm) cut planes are shown in Figs. S5a and S5b, respectively.

In the third case study, similar to CS #2, two PD sources are considered. In this case, though, one of the PD sources (PD3) is located inside the middle cylindrical winding of the transformer, which acts as a barrier for propagation of the electromagnetic fields emitted from PD3. TDoA approaches are clearly not applicable to such a scenario, even with four sensors. The distribution of the y-component of the normalized electric field intensity over the x–z (*y* = 250 mm) is shown in Fig. 6b. Since PD3 is located inside a winding, its focal spot is less pronounced compared to that corresponding to PD1. The distribution of the y-component of the maximum electric field intensity in the *x–y* (*z* = 100 mm) and *y–z* (*x* = 250 mm) cut planes are shown in Figs. S6a and S6b, respectively. The figures show that the proposed method can localize PD1 and PD3 with location errors of 4 mm and 3 mm, respectively. In the next scenario, the Gaussian pulse used to excite the partial discharge PD3 is shifted in time by 4 ns as shown in Fig. S7a. Figure 6c shows the distribution of the y-component of the normalized electric field intensity over the x–z (*y* = 250 mm) cut plane. As can be seen in this figure, the proposed method can localize PD1 and PD3 with the same accuracy. This suggests that a time shift between PD sources does not affect the performance of the proposed method.

In the last scenario, we increase the amplitude of PD3 by a factor of 2 as shown in Fig. S7b. Again, the distribution of the y-component of the normalized electric field intensity over the x–z (*y* = 250 mm) cut plane is shown in Fig. S8. In this figure, the maximum point of the field intensity is located on the PD3 source.

### PD source localization using EMTR: experimental validation

In this section, we provide detailed information on a test setup aimed at experimentally validating the proposed EMTR method to localize PD sources. In the experimental setup, an HP-8753D VNA in the frequency range of 30 kHz-3 GHz is used. The PD sources are represented using a Gaussian pulse with a frequency bandwidth of 300–3000 MHz, which is commonly used in PD studies^6^.

To experimentally locate a PD source using the TR technique, one should take the following three steps:

(i) The electromagnetic waves from the PD source are measured at one location. The measured signal is denoted by *R*(*ω*). This step is referred to as the forward propagation step.
  To perform this step in the frequency domain, we measure the scattering parameter *S*_sp_ using a VNA. The indexes *s* and *p* denote the sensor and PD source, respectively. The response of the sensor can be evaluated as
  1 $$R\left( \omega \right) \, = S_{sp} \left( \omega \right)G\left( \omega \right)$$
  where *ω* is the angular frequency and the capital letters are reserved for the frequency domain parameters. *G*(*ω*) denotes the PD source applied to the monopole antenna. The time domain response, if needed, can be obtained using the inverse fast Fourier transform (iFFT),
  2 $$r\left( t \right) = iFFT\left( {R\left( \omega \right)} \right)$$
(ii) The acquired waveform is time-reversed. In the frequency domain, this corresponds to the complex conjugate operation R*(*ω*), in which * denotes the conjugate operator. In the time domain,
  3 $${\text{iFFT}}\left( {R^{*}\left( \omega \right)} \right) = r\left( { - t} \right)$$
  The time reversed signal is injected back into the medium and measured at a test location, which we will call the guessed location. To perform this, the *S*_xs_(*ω*) parameter between a guessed and the sensor location for the PD source is measured using the VNA in the frequency domain. The index *x* denotes the guessed location. Then, the time reversed response, *x*^TR^(*t*), can be found applying the inverse Fourier transform to
  4 $$\begin{aligned} & X^{TR} \left( \omega \right) \, = S_{xs} \left( \omega \right)R^{*} \left( \omega \right) \\ & \quad = S_{{{\text{xs}}}} \left( \omega \right)\left( {S_{{{\text{sp}}}} \left( \omega \right)G\left( \omega \right)} \right)^{*} \\ \end{aligned}$$
(iii) When the guessed location coincides with the real one, the reciprocity theorem implies that *S*_xs_ = *S*_ps_ = *S*_sp_, and Eq. (4) reduces to
  5 $$X^{{{\text{TR}}}} \left( \omega \right) = \left| {S_{{{\text{sp}}}} \left( \omega \right)} \right|^{{2}} G\left( \omega \right)^{*}$$
  resulting in a maximum peak value of *x*^TR^(*t*) = iFFT(*X*^TR^(*ω*)).

The maximum peak value of *x*^TR^(*t*) over the whole time and for all of the considered guessed locations is used as a criterion to identify the location of the PD source.

It should be noted that the proposed experimental test does not require any information either on the size or on the material of the transformer tank model and its content, since the backpropagation step is carried out experimentally. The above-mentioned procedure can be used to localize any arbitrarily time domain signal.

Due to the practical limitations, a limited number of test points is used in the experiment. It is indeed impossible to measure at all possible locations in space. Note that similar procedures have been used in other studies in the same way we have used them, for testing purposes (see e.g.,^20–22,24^ in the electromagnetic regime and^25–27^ in the acoustic regime). Note, however, that in a practical implementation the backward propagation stage is carried out fully using numerical simulations making all the possible locations accessible.

### Geometry of the problem

**Figure 7:**
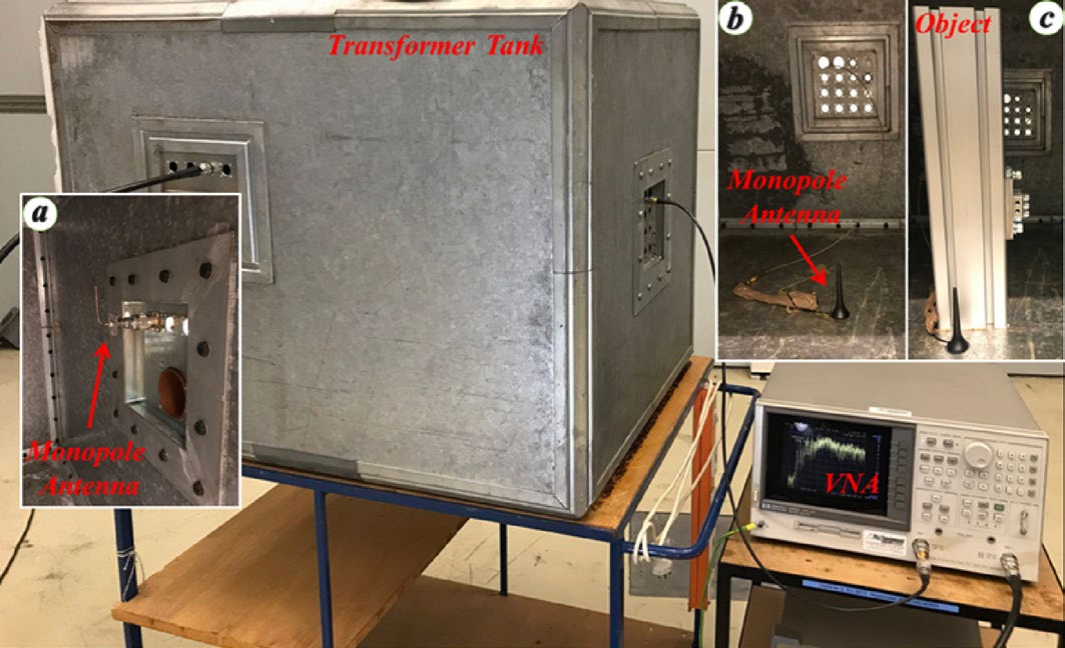
Test setup including the model for the transformer tank and the VNA. (**a**) The monopole antenna used as a sensor, (**b**) the monopole antenna used to emulate the PD source, and (**c**) metallic object representing the transformer winding.

Figure 7 shows pictures of the transformer tank model, including one metallic object used to represent a scatterer inside the transformer, such as, for example, the transformer windings. The size of the transformer tank model is 101 × 73 × 73 cm^3^. The transformer tank and the winding are made of steel and aluminum, respectively. The thickness of the transformer tank model walls is 10 mm. The aluminum object is placed at the center of the transformer tank as shown in Fig. 7c. As mentioned previously, no knowledge of the material, dimensions or geometry is needed since the back-propagation step is carried out experimentally. A 10 mm monopole antenna is used as the PD source that is placed at different locations in the transformer tank model as shown in Fig. 7b. A monopole antenna is also used as the sensor, which was located on the right wall of the transformer tank model as shown in Fig. 7a. The monopole antenna used as the sensor was handmade while the one used to represent the PD source was a commercial monopole commonly used in wireless network communications.

Ideally, we would measure the back-propagated, time-reversed signal at a large number of points in the tank and we would select the point with the maximum peak field. For practical reasons, we selected a limited number of locations and performed back-propagation measurement experiments for each one of them.

**Figure 8:**
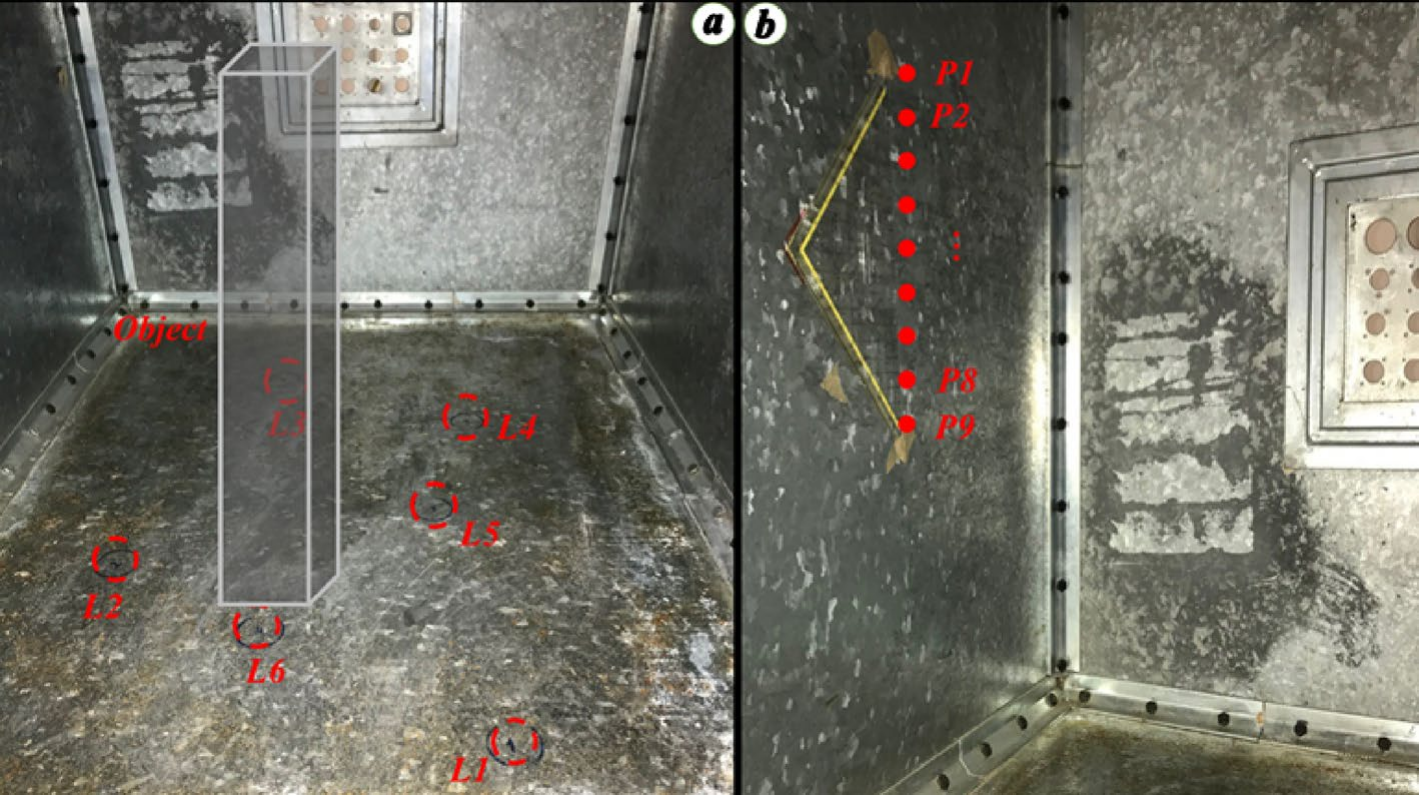
Assumed PD locations. (**a**) On the floor of the tank (*L*_1_, …, *L*_6_). For better illustration, the metallic object was removed temporarily and replaced with a translucid parallelepiped, (**b**) On the left-wall of the transformer tank, *P*_1_, …, *P*_9_ are spaced 2.5 cm from each other.

The assumed PD source locations, labeled *L*_1_ to *L*_6_ on the floor surface of the cavity, can be seen in Fig. 8a. For each experiment, one of these points was chosen as the assumed PD source location and the field resulting from the back-injection of the time-reversed waveform was measured. In selecting points *L*_1_ to *L*_6_, we tried to choose challenging locations. For example, *L*_2_ and *L*_6_ are located close to the scattering object as seen in Fig. 8a. Moreover, the line of sight between *L*_6_ and the sensor is blocked by the object. *L*_6_ is located next to the object (see Fig. 8a) and *L*_2_ behind the object. It is worth noting that, since we have considered only a finite number of test points, the validation cannot be considered as a rigorous proof.

### Comparison of time-domain and frequency-domain measurement results

**Figure 9:**
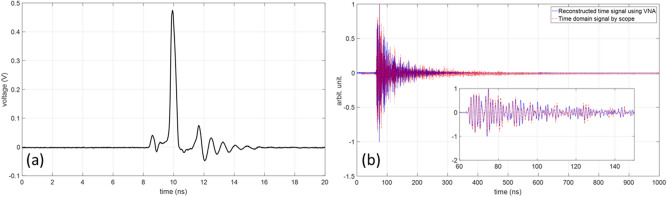
(**a**) The output signal of the pulse generator, (**b**) The dashed-red signal and the solid-blue signal show the impulse response measured at the position *L*_3_ by the 70 GHz scope and reconstructed by the 3 GHz VNA.

In this subsection, we present a preliminary comparison between time-domain and frequency-domain experimental results to confirm the validity of the impulse response. The comparison concerns the impulse response between two points in the cavity under test (in the absence of the object) obtained (*i*) by using a VNA in the frequency domain, and (*ii*) by using an AVI-V-HV1A-B Avtech pulse generator and a DPO77002SX Tektronix oscilloscope with a bandwidth of 70 GHz. In the time-domain experimental setup, a short pulse with a rise time of 0.2 ns was produced by the pulse generator as shown in Fig. 9a. The output of the pulser was connected to the monopole antenna shown in the Fig. 7a. The electromagnetic fields were measured at the different considered locations (*L*_1_ to *L*_6_) inside the cavity using the 70 GHz oscilloscope. This procedure was repeated 50 times and the averaged measured voltages at the input of the receiving antennas were determined. As an example, the measured voltage at *L*_3_ is depicted in Fig. 9b. The reconstructed time-domain signal using the VNA measurements and the directly-measured time-domain signal by the oscilloscope are also shown in this figure. The reconstructed time-domain signal was obtained by multiplying the measured *S*_12_ parameter between the two antennas (the wall-mounted antenna in Fig. 7a and the monopole antenna at *L*_3_), by the Fourier transform of the output pulse of the pulse generator, and evaluating the inverse Fourier transform of the resulting signal As can be seen from Fig. 9b and from the expanded view in the figure inset, there is an excellent agreement between the two results. We can also see from Fig. 9b that the level of noise for the reconstructed signal by the VNA (frequency-domain measurements) is much lower than that of the direct time-domain measured signal.

### Transformer tank model without object

In this subsection, we present a first experimental validation of the EMTR localization technique for the case of an empty tank.

**Figure 10:**
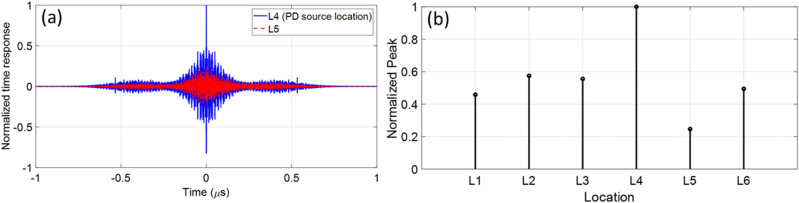
(**a**) Normalized time reversed responses for two guessed locations *L*_4_ and *L*_5_. *L*_4_ is the PD source location. (**b**) The normalized peak values of the time reversed response at the guessed locations *L*_1_ to *L*_6_. The maximum peak occurs at *L*_4_, which corresponds to the PD source location. In this scenario the metallic object is removed.

We considered our PD source to be located at point *L*_4_ (see Fig. 8a). We applied the procedure described in Section II and considered as guessed locations *L*_1_ to *L*_6_. Figure 10a shows the time reversed response for the guessed locations *L*_4_ and *L*_5_. As it can be seen, the peak value of the time reversed response at *L*_4_ (the correct location) is 4 times higher than that at *L*_5_. Figure 10b shows the peak values of the time reversed responses at the guessed locations. It can be seen that the highest peak occurs at the real source location.

### Transformer tank model with object

**Figure 11:**
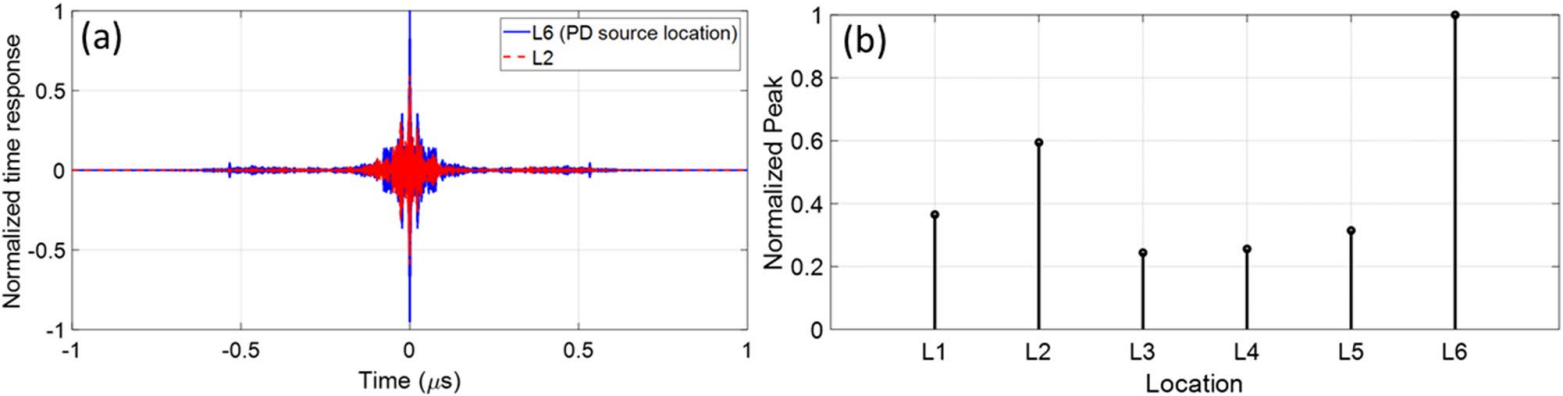
(**a**) Normalized time reversed responses for two guessed locations *L*_2_ and *L*_6_. *L*_6_ is the PD source location. (**b**) The normalized peak values of the time reversed response at the guessed locations *L*_1_ to *L*_6_. The maximum peak occurs at *L*_6_, which corresponds to the PD source location. In this scenario the metallic object is included in the transformer tank model.

To demonstrate the method in the more realistic case in which a winding is present, we include a metallic object inside the transformer tank as shown in Fig. 7c. We considered a PD source located at *L*_6_ near the metallic object as shown in Fig. 8a. We applied again the procedure described in Section II. Figure 11a shows the time reversed response measured at the guessed locations *L*_2_ and *L*_6_. As can be seen, the peak value of the time reversed response at *L*_6_ is 1.8 times higher than that at *L*_2_. Figure 11b shows the peak values of the time reversed responses at the guessed locations, showing that the highest peak occurs at the position of the PD source.

### Accuracy of the TR method

In order to investigate the location accuracy of the proposed method, we considered nine equally spaced PD locations between *P*_1_ and *P*_9_ on the front side wall of the cavity, as shown in Fig. 8b. The distance between two adjacent PD locations is 2.5 cm, which is equal to λ/4 at 3 GHz. In this scenario, the metallic object was removed from the transformer tank model.

**Figure 12:**
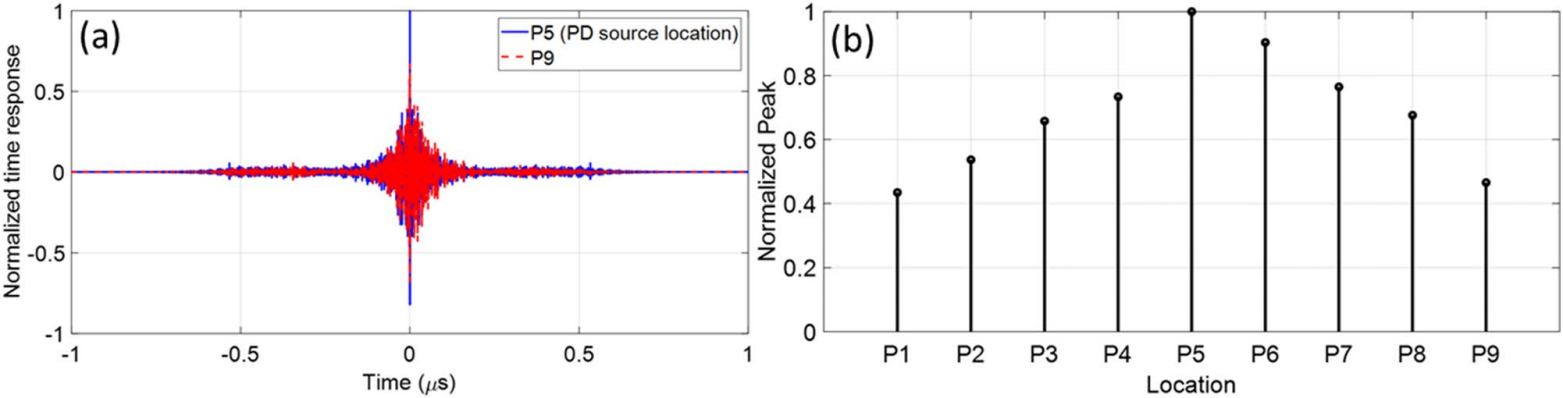
(**a**) Time reversed responses for two guessed locations, *P*_1_ and *P*_9_. In this scenario, the metallic object is removed. The location of *P*_5_ is considered as the ground truth PD source location. (**b**) The normalized peak values of the time reversed response at the guessed locations *P*_1_ to *P*_9_. In this scenario the metallic object is removed. The location of *P*_5_ is considered as the ground truth PD source location.

The position *P*_5_ (the middle point between *P*_1_ and *P*_9_) was considered as the location of the PD source. *P*_1_ to *P*_9_ are considered as guessed locations. One more time, the procedure described in Section II was applied. Figure 12a shows the time reversed response for guessed locations *P*_5_ and *P*_9_, showing that the peak value of the time reversed response for *P*_5_ is 1.5 times larger than that at *P*_9_. Figure 12b shows the peak values of the time reversed responses at all the guessed locations, showing that the proposed method can distinguish the location of the PD source with an accuracy better than 2.5 cm (λ/4). Note that we have also considered other positions (*P*_1_ to *P*_4_ and *P*_6_ to *P*_9_) as the PD location and always obtained the highest peak value of the time reversed response at the PD location. It should be noted that this value for the accuracy is less than the diffraction limit (λ/2), a fact that has been previously reported in the literature as a feature of the TR technique.

## Discussion

Localizing partial discharge (PD) sources is of importance for monitoring and maintenance of power transformers. Time difference of arrival (TDoA) based methods are widely adopted in the literature for the localization of PDs. Recently, TR was suggested as an efficient means to locate PD sources. As opposed to TDoA, which needs at least 4 sensors, TR is in principle able to locate PD sources with only one sensor. Moreover, it does not need line-of-sight wave propagation from PD sources to the sensor and, when only one sensor is used, synchronization is not needed.

In this study, we demonstrated experimentally the ability of the TR process to locate PD sources. A typical TR process includes three steps: (1) recording the PD-emitted field by a sensor, (2) time reversing and back injecting the signal into the medium, (3) using a proper criterion to obtain the focusing point which corresponds to the location of the PD source. In this work, we presented a test setup in which steps one and two are performed experimentally, both in the frequency and in the time domain. The obtained peak electric field value was used as a criterion in the third step. It was found that the accuracy of the proposed method is better than 2.5 cm (λ/4) in a transformer tank model with dimension of 73 × 73 × 103 cm^3^. The effects of the presence of scatterers such as transformer windings were also included in the experimental analysis and found not to affect the location accuracy of the method.

As discussed in^28^, the performance of the EMTR method is not impacted by the PD source (length and polarization) or the presence of noise. Moreover, the presence of the transformer tank acts as a cavity^29^, emulating an infinite number of sensors in the TR method, therefore increasing its performance. In particular, the EMTR method does not require line-of-sight between the PD source and the sensor, as opposed to TDOA-based approaches^19,28^.

However, the geometry of the tank and in particular the presence of the transformer windings has an effect on the localization of PD sources that occur either between two adjacent phase windings when the distance between the outer winding distances is shorter than the minimum wavelength. Furthermore, a degradation in the accuracy of the localization is observed when the PD is located in the immediate vicinity of a metallic plane or object (within λ_min_/2)^19^.

Furthermore, the effect of the presence of environmental white Gaussian noise on the performance of the proposed method has been investigated in^14^. Two values were considered for the signal-to-noise ratio: SNR = 20 dB and SNR = 10 dB. The results show that the proposed method is robust for SNR values greater than 10 dB. Further theoretical and experimental investigations are needed to thoroughly assess the performance of the method. This will be the topic of our future work.

## Supplementary Information

Supplementary Information.

## Data Availability

The data that support the findings of this study are available upon reasonable request from the corresponding author.
