# Supplementary material for: An experimental validation of partial discharge localization using electromagnetic time reversal
Source: Sci Rep. 2021 Jan 8;11:220. doi: 10.1038/s41598-020-80660-z (PMC7794480; doi:10.1038/s41598-020-80660-z)
Supplement: Supplementary file 1 — Supplementary Information. [file 41598_2020_80660_MOESM1_ESM.docx]

**Supplementary Information**

**An Experimental Validation of Partial Discharge Localization Using Electromagnetic Time Reversal**

Hamidreza Karami^1^, Mohammad Azadifar^2^, Marcos Rubinstein^2^, Farhad Rachidi^1^

^1^Electromagnetic Compatibility Laboratory, Swiss Federal Institute of Technology (EPFL), Lausanne, Switzerland

^2^Institute for Information and Communication Technologies, University of Applied Sciences of Western Switzerland (HES-SO), Yverdon-les-Bains, Switzerland

Corresponding author:

Farhad Rachidi

ELL 138, ELL building, Station 11

1015 Lausanne, Vaud, Switzerland

Tel. +41 21 693 2620

Fax. +41 21 693 2661

farhad.rachidi@epfl.ch

**Contents of this file**

The supplementary information contains 8 figures.

Figures

Fig. S1 The Gaussian pulses used to model PD sources with bandwidth of 1, 2, and 3 GHz.

Fig. S2 The location of the assumed (black filled circles) and estimated (red crosses) PD sources for the 2D transformer model shown in Fig. 1. The number of Monte-Carlo simulations is 100. The blue lines show the location error between the actual and the estimated PD sources.


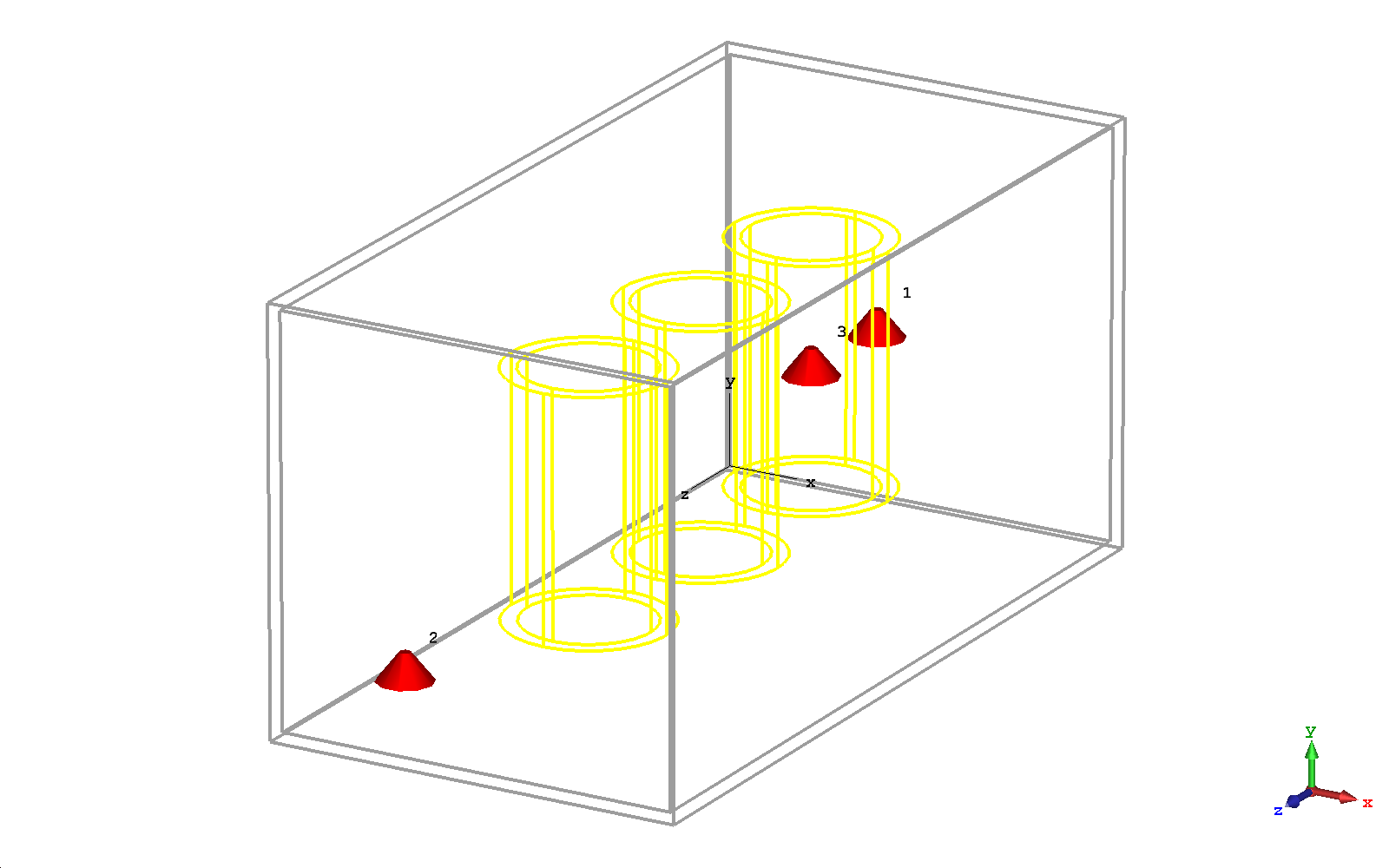


Fig. S3 Geometry of the 3D power transformer wireframe model designed in the CST-MWS software. The cones show the ports used as a sensor (port#2) and as the PD sources (port#1 and port#3).


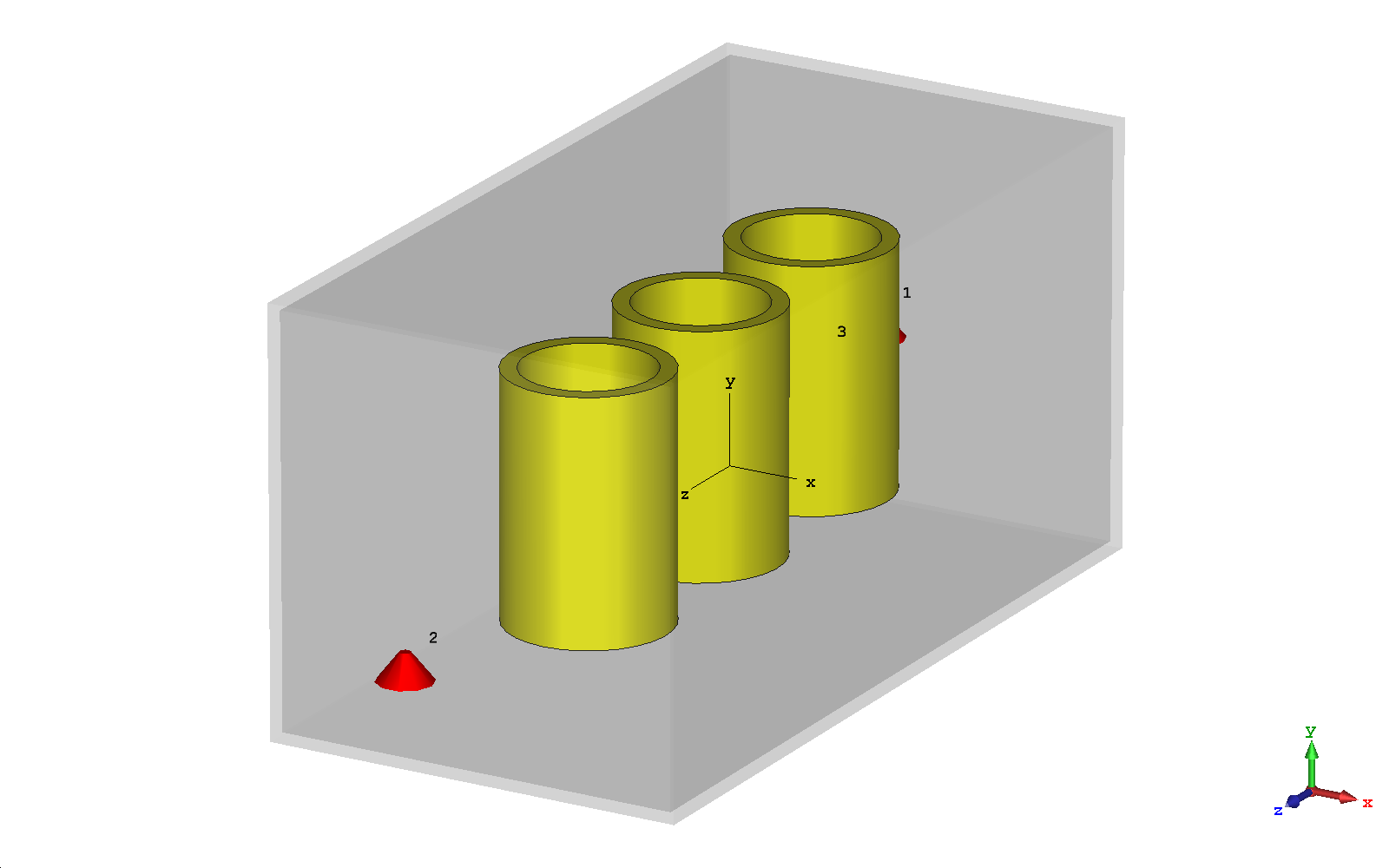


Fig. S4 Geometry of the 3D power transformer solid model designed in the CST-MWS software.

Fig. S5a Distribution of the normalized maximum y-component of the electric field intensity in the *x-y* cut plane (*z* = 100 mm, CS #2). The blue circles and black cross show the actual location of the PD sources and the maximum of the electric field intensity.

Fig. S5b Distribution of the normalized maximum y-component of the electric field intensity in the *y-z* cut plane (*x* = 250 mm, CS #2). The blue circles and black cross show the actual location of the PD sources and the maximum of the electric field intensity.

Fig. S6a Distribution of the normalized maximum y-component of the electric field intensity in the *y-z* cut plane (*x* = 250 mm, CS #3). The blue circles and black cross show the actual location of the PD sources and the maximum of the electric field intensity.

Fig. S6b Distribution of the normalized maximum y-component of the electric field intensity in the *x-y* cut plane (*z* = 100 mm, CS #3). The blue circles and black cross show the actual location of the PD sources and the maximum of the electric field intensity.

Fig. S7a The black solid line shows a Gaussian pulse (g(t)) with a 3 GHz bandwidth used to model the PD1 source in CS#3. The red dashed line shows the shifted Gaussian pulse (g(t-4e-9)) with a 3 GHz bandwidth used to model the PD3 source in CS#3. See the results shown in Fig. 6c.

Fig. S7b The black solid line shows a Gaussian pulse (g(t)) with a 3 GHz bandwidth used to model the PD1 source in CS#3. The red dashed line shows the shifted Gaussian pulse (g(t-4e-9)) with two times the amplitude of original pulse and a 3 GHz bandwidth used to model the PD3 source in CS#3. See the results shown in Fig. S8.

Fig. S8 Distribution of the normalized maximum y-component of the electric field intensity in the *x-z* cut plane (CS #3). The blue circles and black cross show the actual location of the PD sources and the maximum of the electric field intensity. In this simulation, PD1 and PD3 are excited by a Gaussian and a shifted Gaussian pulse shown in Fig. S7b.
